# The association of systemic inflammatory biomarkers with non-alcoholic fatty liver disease: a large population-based cross-sectional study

**DOI:** 10.1016/j.pmedr.2023.102536

**Published:** 2023-12-07

**Authors:** Wu Sun, Yan Fang, Bing Zhou, Guoliang Mao, Jiao Cheng, Xinxin Zhang, Yinhua Liu, Hao Chen

**Affiliations:** a Department of Pathology, Yijishan Hospital, The First Affiliated Hospital of Wannan Medical College, Wuhu, Anhui 241000, China; b Department of Criminal Science and Technology, Shandong Police College, Jinan, Shandong 250014, China; c Department of Pathology, School of Basic Medical Sciences, Wannan Medical College, Wuhu, Anhui 241002, China; d Postdoctoral Research Station of Clinical Medicine, Jinan University, Guangzhou, Guangdong 510632, China

**Keywords:** Cross-sectional study, Systemic immune-inflammation index, Non-alcoholic fatty liver disease, System inflammation response index, United States

## Abstract

**Purpose:**

The aim of this study was to explore the relationship between non-alcoholic fatty liver disease (NAFLD) and the two blood inflammatory markers including the systemic immune-inflammation (SII) index, and the system inflammation response index (SIRI).

**Methods:**

The National Health and Nutrition Examination Survey data between the year of 2017–2018 was used for this cross-sectional study. In order to analyze the association of SII index, and SIRI and risk of NAFLD, we used multivariable logistic regression models, restricted cubic spline (RCS) plot, and subgroup analysis to analyze the data.

**Results:**

In total, there were 1,199 individuals who participated in the survey. As shown by the RCS plot, SII index, and SIRI were linked with NAFLD risk in a U-shaped pattern. With regard to known confounding variables, when comparing the lowest quartile, the odds ratio with 95 % confidence interval for prevalence of NAFLD across the quartiles of SII index and SIRI were (0.89 (0.57, 1.41), 0.56 (0.35, 0.89) and 1.01 (0.64, 1.59)), and (0.77 (0.48, 1.23), 0.79 (0.50, 1.24) and 0.94 (0.60, 1.47)), respectively. Additionally, SII index, and SIRI and NAFLD risk also were U-curve correlated among the participants in age ≥60 years, female, without hypertension, and BMI of ≥30 kg/m^2^.

**Conclusions:**

There was a U-shaped association of SII index and SIRI with prevalence of NAFLD, indicating that SII index and SIRI should be monitored dynamically.

## 1 Introduction

Non-alcoholic fatty liver disease (NAFLD) still occupies an important place in adult chronic liver diseases, with a global prevalence estimated at 32.4 % and rising (Riazi et al., 2022). NAFLD refers to liver fat deposition and hepatocyte steatosis caused by metabolic disorders in addition to alcohol, immunity, infection and other factors, and thus develops into liver inflammation, fibrosis, cirrhosis and even liver cancer (Zafrani, 2004). Although its mechanism has not been clearly explained, it is associated with lipid metabolism disorder, insulin resistance decline, type 2 diabetes and so on (Yan et al., 2022).

The blood inflammatory index is an inexpensive and easily accessible biomarker. As an indicator of both local immune response and systemic inflammation, the SII index, integrating three of inflammatory cells (lymphocyte, neutrophil, and platelet), is considered to be a good and stable index (Hu et al., 2014, Tong et al., 2017). Several studies have shown that SII index can predict the outcome of patients with multiple cancers, acute ischemic stroke, heart failure, and acute kidney injuries (Jiang et al., 2022, Tang et al., 2021, Xie et al., 2021, Yang et al., 2018, Zhou et al., 2022). Additionally, SIRI, which is composed of lymphocyte, monocyte, and neutrophil counts, is a more comprehensive indicator of chronic low-grade inflammation (Jia et al., 2019). The previous studies suggested that SIRI has been widely recognized as the potential indicators for early diagnosis and prognosis monitoring in stroke, inflammatory diseases and cancers (Li et al., 2017, Ma et al., 2022, Qi et al., 2016, Zhang et al., 2021). The consideration of the harmful effects of NAFLD, the identification of risk factors, and the taking of measures to prevent or control the consequences, as soon as possible, is very helpful in reducing or preventing the consequences of NAFLD. There has been no evidence that the SII index and SIRI are associated with NAFLD risk in the general American population based on the epidemiological research to date. By analysing the data from the Nutrition and Health Examination Survey (NHANES) between the year of 2017–2018, we tried to examine the association of the SII index and SIRI with the prevalence of NAFLD.

## 2 Methods

### 2.1 Study population

**Fig. 1:**
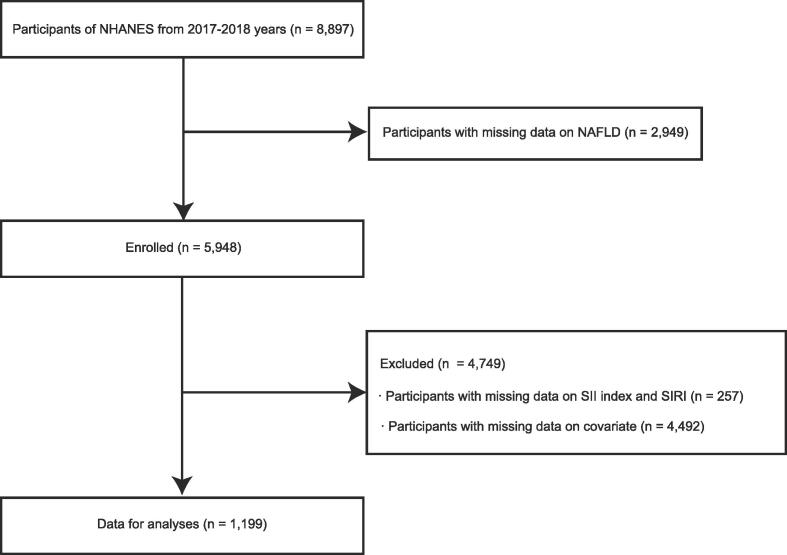
Study flow chart. Abbreviations: NAFLD, non-alcoholic fatty liver disease; SII index, systemic immune-inflammation index; NHANES, National Health and Nutrition Examination Surveys; SIRI, system inflammation response index;

For the purpose of collecting information about the general population's health and nutrition, the National Health and Nutrition Examination Survey (NHANES) database (https://www.cdc.gov/nchs/nhanes/) uses a multistage stratified random sampling method (Xiao et al., 2023a). For the purpose of this study, we analysed the data from the NHANES data from the 2017 and 2018 years. In total, 8,897 participants in total sample were included in the study, however we excluded 2,949 participants without NAFLD data, and 257 participants without SII index and SIRI data. Further, participants who did not have data on missing covariate data (n = 4,492) were also excluded from the analysis. Finally, the research included 1,199 individuals (Fig. 1). Every participant was required to provide informed consent prior to participating in the study. In addition, the National Center for Health Statistics obtained institutional review board approval prior to data collection (Zipf et al., 2013).

### 2.2 Covariates

In the study, the covariates were as follows: age, education level (less than high school, more than high school, and high school), sex (male and female), family poverty income ratio (PIR), marital status (no partner, having a partner and unmarried), smoker (no, former, now), race/ethnicity (Other Race, Mexican American, Non-Hispanic Black, Other Hispanic and Non-Hispanic White), the complication of angina pectoris, hypertension, coronary heart disease (CHD), diabetes mellitus (DM), heart attack, congestive heart failure (CHF), and stroke, drinker (never, mild, moderate, heavy), body mass index (BMI), fast glucose (FBG), waist circumference, hemoglobin (Hb), alanine aminotransferase (ALT), high-sensitivity C-reactive protein (hs CRP), aspartate amino transferase (AST), blood urea nitrogen (BUN), gamma-glutamyl transpeptidase (GGT), uric acid (UA), high-density lipoprotein-cholesterol (HDL-C), estimated glomerular filtration rate (eGFR), total cholesterol (TC), serum creatinine (Scr), and triglyceride (TG). A detailed explanation of the variables involved in this study can be found at the following link: www.cdc.gov/nchs/nhanes/.

### 2.3 Calculation of the SII index

In the study, the blood samples were collected from the participants who were fasted in the morning. The professional technicians used the automated hematology analyzing devices (Coulter® DxH 800 analyzer) to measure blood count (monocyte, lymphocyte (Lym), platelet counts and neutrophil (Neu)). We calculated SII index and SIRI according to the following formula for each participant:

SIRI (×10^9^/L) = neutrophil count (×10^9^/L) × monocyte(×10^9^/L)/lymphocyte count (×10^9^/L) (Qi et al., 2016).

SII index (×10^9^/L) = neutrophil count (×10^9^/L)/lymphocyte count (×10^9^/L) × platelet count (×10^9^/L) (Hu et al., 2014, Xiao et al., 2023b).

### 2.4 NAFLD measurement

NAFLD was defined using the U.S. fatty liver index (FLI), a well-validated diagnostic index (Ruhl and Everhart, 2015), which was employed utilizing NHANES III data and calculated as an equation according to the previous study (Bedogni et al., 2006, Kobyliak et al., 2018) that included information on GGT, BMI, TG, and waist circumference. All the information was collected concurrently with the status of iron metabolism. NAFLD was defined as an FLI score of ≥60. The FLI formula is expressed as follows:

**FLI = (e^0.953∗ln^**^(TG)+0.139∗BMI+0.718∗ln (GGT)+0.053∗waist circumference−15.745^)/(1 + e^0.953∗ln (TG)+0.139∗BMI+0.718∗ln (GGT)+0.053∗waist circumference−15.745^) ∗ 100 (Zhang et al., 2022).

### 2.5 Statistical analysis

In this study, we conducted all analyses by using the SPSS version 23.0 (SPSS Inc., Chicago, IL, USA) and R version 4.3.2 (R Foundation for Statistical Computing, Vienna, Austria). The estimates were based on the weighted NHANES sample. The statistical significance of the study was determined by *P*-value < 0.05. The two systemic inflammatory biomarkers were all divided into quartiles, including SII index (Q1, 50.000–291.652; Q2, 292.653–423.059; Q3, 423.060–603.132; Q4, 603.133–3250.715;) and SIRI (Q1, 0.120–0.632; Q2, 0.633–0.924; Q3, 0.925–1.371; Q4, 1.372–10.890;), and the lowest quartile (Q1) of SII and SIRI served as the reference group (Q1). Variables that are continuous are expressed as means (standard deviations, SDs), while variables that are categorical are expressed as percentages (%). We used weighted Student’s *t*-test for continuous variables and weighted chi-square tests for categorical variables to calculate differences between groups. The association of SIRI, and SII index with prevalence of NAFLD were investigated using weighted multivariate logistic regression analysis. Firstly, model 1 was adjusted for sex, race/ethnicity, and age. Secondly, model 2 was adjusted for model 1 variables plus the complication of hypertension, smoke status, marital status, drink status, education level, the complication of DM, family PIR, and BMI. Finally, model 3 was adjusted for model 2 variables plus the complication of CHF, CHD, stroke, angina pectoris, and heart attack, ALT, TG, hs CRP, GGT, mean energy intake, UA, BUN, AST, waist circumference, FBG, Scr, Hb, eGFR, TC, and HDL-C.

## 3 Results

### 3.1 Baseline characteristics

Table 1 provides an overview of the laboratory examinations and basic clinical characteristics. There was a prevalence of 28.0 % of NAFLD in this study. We computed that the number of participants in this study is likely representative of the total U.S. population of 64,397,408. There was a statistically significant difference in age, sex Marital status, hypertension, DM, alcohol user, CHF, angina pectoris, BMI, Neu, mean energy intake, GGT, Lym, waist circumference, Ast, platelet, Hb, Alt, hs CRP, and eGFR between non-NAFLD and NAFLD.

**Table 1:** Demographic characteristics of the study adults in the United States from NHANES 2017–2018 (n = 1,199).

Variables	Overall (n = 1,199)	Non-NAFLD (n = 863)	NAFLD (n = 336)	*P*-value
Age, years	45.87±0.85	44.02±0.87	51.24±1.60	0.001

Sex, n (%)				0.002
Male	585 (48.8)	467 (38.9)	118 (9.8)	
Female	614 (51.2)	396 (33.0)	218 (18.2)	

Race, n (%)				0.533
Mexican American	165 (13.8)	110 (9.2)	55 (4.6)	
Other Hispanic	108 (9.0)	82 (6.8)	26 (2.2)	
Non-Hispanic Black	288 (24.0)	209 (17.4)	79 (6.6)	
Non-Hispanic White	430 (35.9)	302 (25.2)	128 (10.7)	
Other race	208 (17.3)	160 (13.3)	48 (4.0)	
Family PIR	3.22±0.09	3.25±0.08	3.14±0.18	0.521

Education level, n (%)				0.414
Less than high school	172 (14.3)	118 (9.8)	54 (4.5)	
High school	276 (23.0)	188 (15.7)	88 (7.3)	
More than high school	751 (62.6)	557 (46.5)	194 (16.2)	

Marital status, n (%)				0.009
Having a partner	724 (60.4)	502 (41.9)	222 (18.5)	
No partner	257 (21.4)	181 (15.1)	76 (6.3)	
Unmarried	218 (18.2)	180 (15.0)	38 (3.2)	

Hypertension, n (%)				0.002
No	709 (59.1)	537 (44.8)	172 (14.3)	
Yes	490 (40.9)	326 (27.2)	164 (13.7)	

DM, n (%)				0.007
No	966 (80.6)	716 (59.7)	250 (20.9)	
Yes	233 (19.4)	147 (12.3)	86 (7.2)	

Smoker, n (%)				0.561
No	705 (58.8)	495 (41.3)	210 (17.5)	
Former	270 (22.5)	195 (16.3)	75 (6.3)	
Now	224 (18.7)	173 (14.4)	51 (4.3)	

Alcohol user, n (%)				< 0.001
No	117 (9.9)	51 (4.3)	66 (5.5)	
Mild	560 (46.7)	425 (35.4)	135 (11.2)	
Moderate	259 (21.6)	185 (15.4)	74 (6.2)	
Heavy	263 (21.9)	202 (16.8)	61 (5.1)	

CHD, n (%)				0.131
No	1160 (96.7)	842 (70.2)	318 (26.5)	
Yes	39 (3.3)	21 (1.8)	18 (1.5)	

CHF, n (%)				0.013
No	1181 (98.5)	850 (70.9)	331 (27.6)	
Yes	18 (1.5)	13 (1.1)	5 (0.4)	

Angina pectoris, n (%)				0.048
No	1177 (98.2)	850 (70.9)	327 (27.3)	
Yes	22 (1.8)	13 (1.1)	9 (0.8)	

Heart attack, n (%)				0.050
No	1159 (96.7)	836 (69.7)	323 (26.9)	
Yes	40 (3.3)	27 (2.3)	13 (1.1)	

Stroke, n (%)				0.611
No	1162 (96.9)	839 (70.0)	323 (26.9)	
Yes	37 (3.1)	24 (2.0)	13 (1.1)	
BMI, kg/m^2^	29.24±0.37	28.72±0.36	30.73±0.77	0.019
Waist circumference, cm	99.56±0.82	98.10±0.81	103.78±1.69	0.005
Mean energy	2141.06±30.69	2164.97±36.09	2071.52±30.60	0.022

Intake (kcal/day)				
Neu, 1000 cells/uL	3.85±0.07	3.78±0.08	4.04±0.11	0.038
Lym, 1000 cells/uL	2.06±0.03	2.03±0.04	2.15±0.04	0.044
Monocyte, 1000 cells/uL	0.54±0.01	0.54±0.01	0.54±0.02	0.888
Platelet, 10^9/L	238.03±3.28	234.17±3.52	249.24±5.27	0.012
Hb, g/dL	14.44±0.08	14.58±0.09	14.03±0.12	0.001
FBG, mg/dL	108.89±1.43	107.27±1.00	113.61±3.56	0.078
Hs CRP, mg/L	3.37±0.23	2.83±0.16	4.95±0.76	0.018
Alt, U/L	23.88±0.79	26.82±1.09	15.33±0.52	< 0.001
Ast, U/L	22.76±0.63	24.69±0.84	17.14±0.33	< 0.001
GGT, iu/L	29.25±1.18	31.52±1.63	22.64±0.95	< 0.001
TC, mg/dL	187.43±2.19	186.53±2.39	190.07±3.19	0.307
TG, mg/dL	108.94±3.39	108.20±4.42	111.10±4.08	0.649
HDL, mg/dL	54.78±0.79	54.86±0.84	54.55±1.13	0.780
BUN, mg/dL	14.49±0.20	14.34±0.17	14.93±0.41	0.137
UA, mg/dL	5.41±0.06	5.40±0.08	5.46±0.08	0.596
Scr, mg/dL	0.86±0.01	0.87±0.01	0.83±0.01	0.111
eGFR, ml/min/1.73 m^2^	96.93±1.11	98.20±1.25	93.26±1.31	0.007
SII index	475.65±11.45	463.97±12.28	509.63±18.88	0.033
SIRI	1.11±0.03	1.10±0.03	1.13±0.05	0.619

Abbreviations: DM, diabetes mellitus; CHD, coronary heart disease; CHF, congestive heart failure; BMI, body mass index; Hb, hemoglobin; FBG, fast glucose; Hs CRP, hypersensitive C-reactive protein; Alt, alanine aminotransferase; Ast, aspartate amino transferase; GGT, gamma glutamyl transferase; Alk, alkaline phosphatase; TC, total cholesterol; TG, triglycerides; HDL-cholesterol, high density lipoprotein-cholesterol; BUN, blood urea nitrogen; UA, uric acid; Scr, serum creatinine; eGFR, estimated glomerular filtration rate; SII index, systemic immune inflammation index; SIRI, system inflammation response index.

### 3.2 Association of SII index and SIRI with NAFLD

**Fig. 2:**
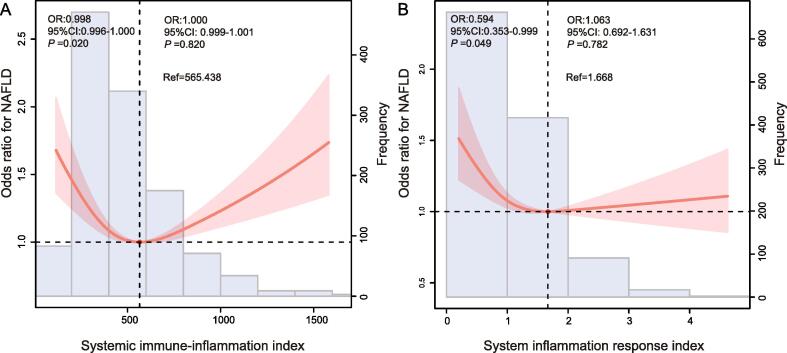
The restricted cubic spline curve for the association of (a) SII index and (b) SIRI with the prevalence of NAFLD in adults in the United States from NHANES 2017–2018. Abbreviations: OR, odd ratio; CI, confidence interval; SII index, systemic immune-inflammation index; NAFLD, non-alcoholic fatty liver disease; SIRI, system inflammation response index.

Based on the restricted cubic spline plot (RCS), we can notice that there is a U-shaped association between SII index and SIRI coexisting with NAFLD prevalence (*P* for nonlinearity < 0.05, Fig. 2A and B). With the increase of SII index and SIRI, NAFLD risk was significantly reduced. The prevalence of NAFLD was lowest when the SII index and SIRI reached 565.438 and 1.668, respectively, and then the curve showed an upward trend. The association of SII index and SIRI with prevalence of NAFLD was investigated employing three multivariate logistic regression models (Models 1, 2, and 3) (Table 2, Table 3). With regard to known confounding variables, when comparing the lowest quartile, the odds ratio with 95 % confidence interval for prevalence of NAFLD across the quartiles of SII index and SIRI were (0.89 (0.57, 1.41), 0.56 (0.35, 0.89) and 1.01 (0.64, 1.59)), and (0.77 (0.48, 1.23), 0.79 (0.50, 1.24) and 0.94 (0.60, 1.47)), respectively.

**Table 2:** The adjusted ORs for the association of SII index with the prevalence of NAFLD in adults in the United States from NHANES 2017–2018.

SII index	Model 1	Model 2	Model 3
	OR (95 %CI)	OR (95 %CI)	OR (95 %CI)
Q1	Ref.	Ref.	Ref.
Q2	0.98 (0.67, 1.44)	0.97 (0.71, 1.61)	0.89 (0.57, 1.41)
Q3	0.85 (0.58, 1.26)	0.75 (0.50, 1.14)	0.56 (0.35, 0.89) *
Q4	1.74 (1.21, 2.51) **	1.58 (1.07, 2.34) *	1.01 (0.64, 1.59)
*P* for trend	0.006	0.071	0.668

Abbreviations: SII index, systemic immune inflammation index; NAFLD, non-alcoholic fatty liver disease; Q1, 50.000–291.652; Q2, 292.653–423.059; Q3, 423.060–603.132; Q4, 603.133–3250.715; *P < 0.05; **P < 0.01; OR, odd ratio; CI, confidence interval. Model 1: age, sex and race/ethnicity. Model 2: model 1 variables plus education level, marital status, family poverty income ratio, the complication of hypertension, and diabetes mellitus, smoke status, drink status, and body mass index. Model 3 was adjusted for model 2 variables plus the complication of coronary heart disease, congestive heart failure, angina pectoris, heart attack, and stroke, mean energy intake, waist circumference, fast glucose, hemoglobin, high-sensitivity C-reactive protein, alanine aminotransferase, aspartate amino transferase, gamma-glutamyl transpeptidase, high-density lipoprotein-cholesterol, total cholesterol, triglyceride, blood urea nitrogen, uric acid, serum creatinine, and estimated glomerular filtration rate.

**Table 3:** The adjusted ORs for the association of SIRI with the prevalence of NAFLD in adults in the United States from NHANES 2017–2018.

SIRI	Model 1	Model 2	Model 3
	OR (95 %CI)	OR (95 %CI)	OR (95 %CI)
Q1	Ref.	Ref.	Ref.
Q2	0.88 (0.60, 1.28)	0.90 (0.60, 1.34)	0.77 (0.48, 1.23)
Q3	1.04 (0.72, 1.52)	0.91 (0.61, 1.36)	0.79 (0.50, 1.24)
Q4	1.13 (0.77, 1.64)	0.96 (0.64, 1.45)	0.94 (0.60, 1.47)
*P* for trend	0.379	0.895	0.214

Abbreviations: SIRI, system inflammation response index; NAFLD, non-alcoholic fatty liver disease; Q1, 0.120–0.632; Q2, 0.633–0.924; Q3, 0.925–1.371; Q4, 1.372–10.890; OR, odd ratio; CI, confidence interval. Model 1: age, sex and race/ethnicity. Model 2: model 1 variables plus education level, marital status, family poverty income ratio, the complication of hypertension, and diabetes mellitus, smoke status, drink status, and body mass index. Model 3 was adjusted for model 2 variables plus the complication of coronary heart disease, congestive heart failure, angina pectoris, heart attack, and stroke, mean energy intake, waist circumference, fast glucose, hemoglobin, high-sensitivity C-reactive protein, alanine aminotransferase, aspartate amino transferase, gamma-glutamyl transpeptidase, high-density lipoprotein-cholesterol, total cholesterol, triglyceride, blood urea nitrogen, uric acid, serum creatinine, and estimated glomerular filtration rate.

### 3.3 Subgroup analyses

Using subgroup analyses stratified based on age, hypertension, sex, DM, and BMI, we investigated the relationship between SII index as well as SIRI and NAFLD risk in various populations (Supplementary Figs. 1 and 2; Supplementary Tables 1 and 2). The stratified analysis revealed that SII index, and SIRI and NAFLD risk also were U-curve correlated among participants in age ≥ 60 years, female, without hypertension, and BMI of ≥30 kg/m^2^.

## 4 Discussion

In the study, firstly, we found that a U-shaped correlation between the SII index and prevalence of NAFLD in the RCS plot. The SII index is a marker of the comprehensive evaluation system and is derives from peripheral neutrophils, lymphocytes, and platelets counts, which probably indicate three pathways, such as the formation of inflammation, thrombus formation, and adaptive immunity, that contribute to the disease process (Azab et al., 2010, Kurtul and Ornek, 2019). It is well known that inflammation plays an important role in the occurrence and progression of NAFLD, which can lead to a change in the levels of peripheral blood leukocytes as a result of such a process (Tilg et al., 2021). Additionally, it is important to point out that the presence of the neutrophils in the liver is a hallmark of inflammation that can occur in the several different types of liver disease. A common feature of NAFLD is the presence of neutrophil infiltration, which results in the recruitment of macrophages and cell damage resulting from the release of inflammatory mediators or reactive oxygen species (Rensen et al., 2009). A systemic inflammatory condition will result in an increase in the total circulating neutrophils and platelets, but a decrease in lymphocytes. Previous studies have shown that hepatic stellate cells were shown to increase the survival of neutrophils, which, in turn, contributes to the formation of reactive oxygen species in the liver, which will lead to liver fibrosis (Zhou et al., 2018). The platelets interplay with inflammatory cells, which results in the release of chemokines, which promotes the accumulation of immune mediators, which plays a crucial role in promoting the formation of NAFLD (Ye et al., 2023). Song et al. found that a positive correlation was found between SII index and the hepatic steatosis among U.S. adults. Thus, the SII index may be an effective and affordable tool for detecting hepatic steatosis in a simple and straightforward manner (Song et al., 2022). This is a good explanation for the increased risk of NAFLD as SII increases. Zhao et al. also showed that in population with NAFLD identified by ultrasound, SII index had a J-shaped curve associated with all-cause deaths and higher SII levels were associated with increased mortality. Despite the fact that multiple confounding variables were adjusted for, this association still persisted (Zhao et al., 2023). This conclusion is consistent with the findings of the study. Additionally, this study is the first to find a correlation between SIRI and NAFLD. We also found a U-shaped correlation between the SIRI and prevalence of NAFLD. As mentioned above, inflammation plays a crucial role in the development and progression of NAFLD. Zhao et al. revealed that as the level of continuous SIRI increases by a unit, there is a 30 % increase in the hazard of mortality due to all causes as well as cardiovascular disease among hypertensive patients (Zhao et al., 2022). Additionally, a few studies have shown that in certain types of inflammation-related disorders like acne vulgaris, and chronic spontaneous urticaria, SIRI is with the potential to reliably predict the effectiveness of anti-inflammatory therapies (Coşansu et al., 2022, Cosansu et al., 2022). However, broader investigations are necessary to explore the relationship between SIRI and prevalence of NAFLD.

This study has strong strengths in terms of its large sample size and representative sample selection. As a result, the findings are generalizable. It is important to note, however, that our study has several limitations that should be taken into account. Firstly, due to year limitations, we only included general population data from NHANES 2017–2018. Secondly, the determination of some covariates was derived from self-report questionnaires, so self-reported confounders would emerge. Thirdly, we could not rule out the possibility that NAFLD may be caused by drug-induced hepatotoxicity. Due to this, we cannot determine a causal relationship between a history of drug use and NAFLD. Finally, due to the fact that this was a cross-sectional study, it was limited to identifying the associations rather than causal relationships.

## 5 Conclusion

To conclude, there is a U-shaped curve in the American population when it comes to the SII index, and SIRI in relation to the prevalence of NAFLD. The SII index or SIRI can serve as a biomarker for NAFLD and a diagnostic or therapeutic target for the systemic inflammation. The potential mechanisms underlying the systemic inflammatory biomarkers (SII index and SIRI), in relation to NAFLD need to be further explored.

## Author contributions

Wu Sun, and Yan Fang contributed to hypothesis development and manuscript preparation. Bing Zhou, and Guoliang Mao contributed to the study design. Wu Sun, Yan Fang, Jiao Cheng, and Xinxin Zhang undertook data analyses. Yinhua Liu contributed to the conception and design of the work. Hao Chen supervised the study and wrote the manuscript. All authors approved the final draft of the manuscript for publication.

## Declaration of competing interest

The authors declare that they have no known competing financial interests or personal relationships that could have appeared to influence the work reported in this paper.

## Data Availability

Data will be made available on request.
