# Supplementary figures and images for: The association of systemic inflammatory biomarkers with non-alcoholic fatty liver disease: a large population-based cross-sectional study
Source: Prev Med Rep. 2023 Dec 7;37:102536. doi: 10.1016/j.pmedr.2023.102536 (PMC10767190; doi:10.1016/j.pmedr.2023.102536)

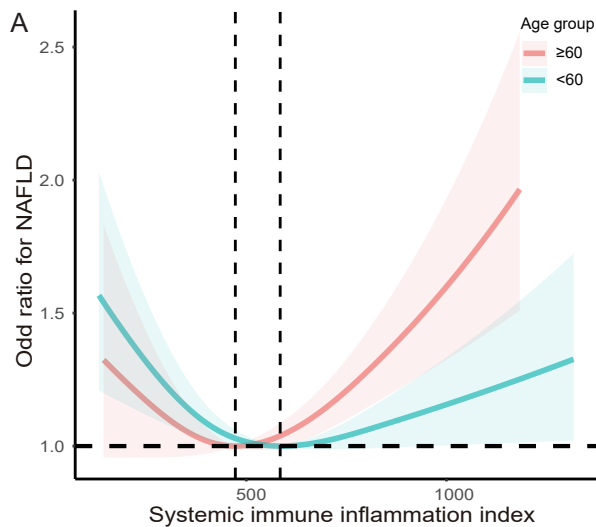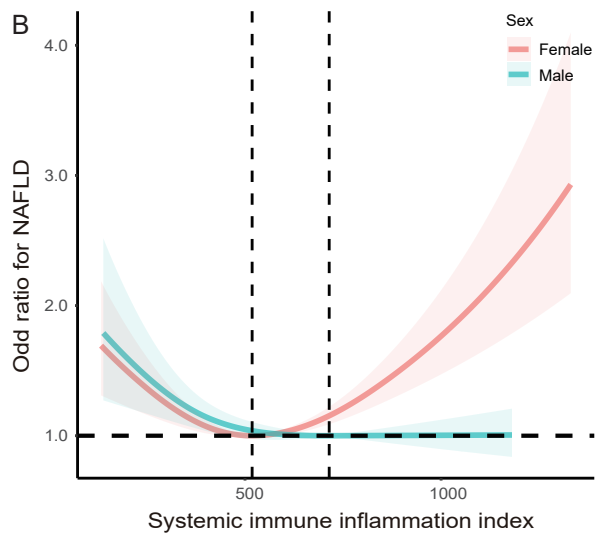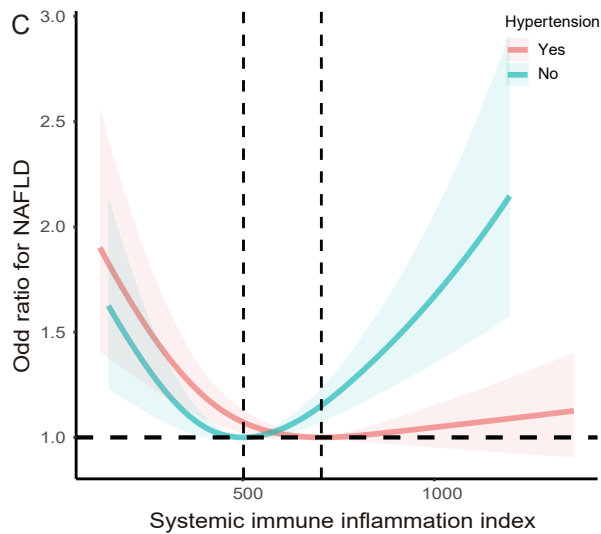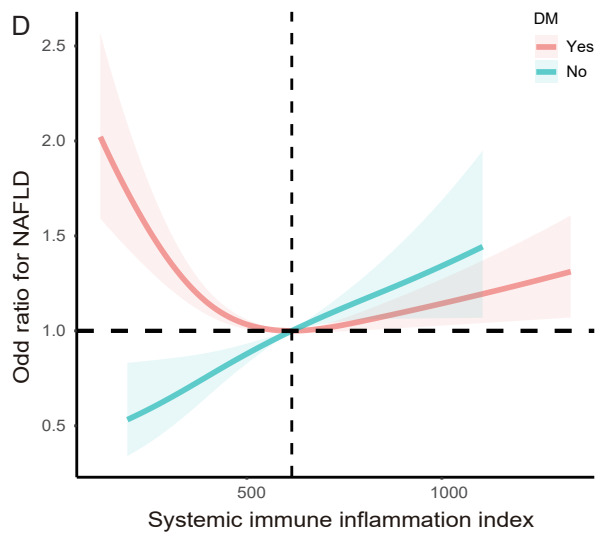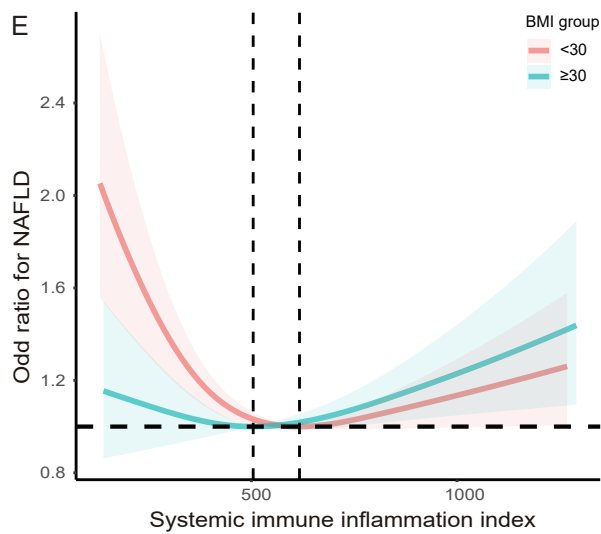

Supplement: Supplementary data 1 [file mmc1.pdf]

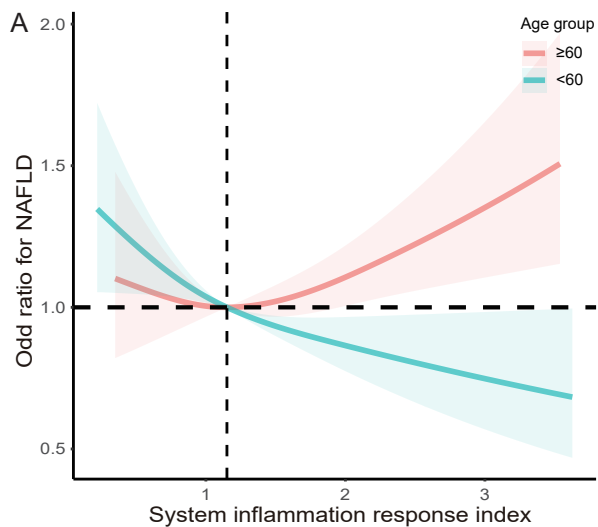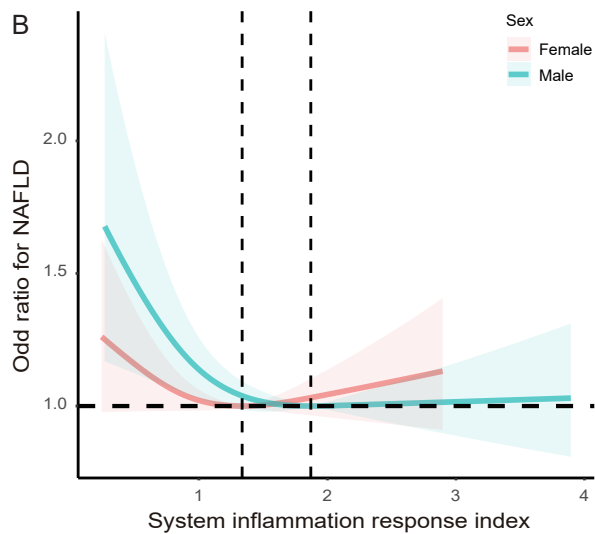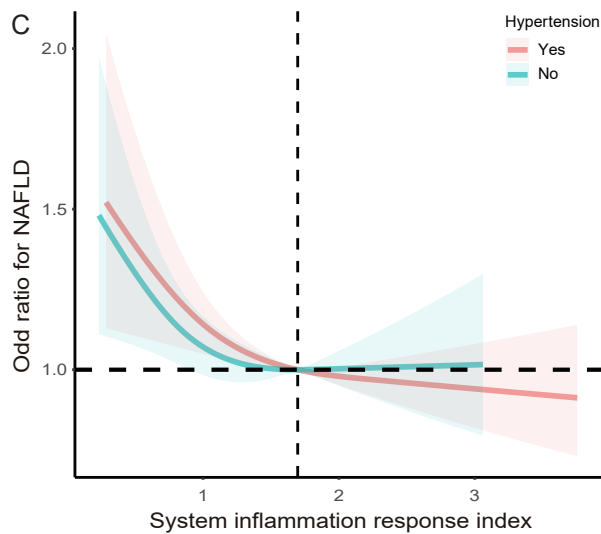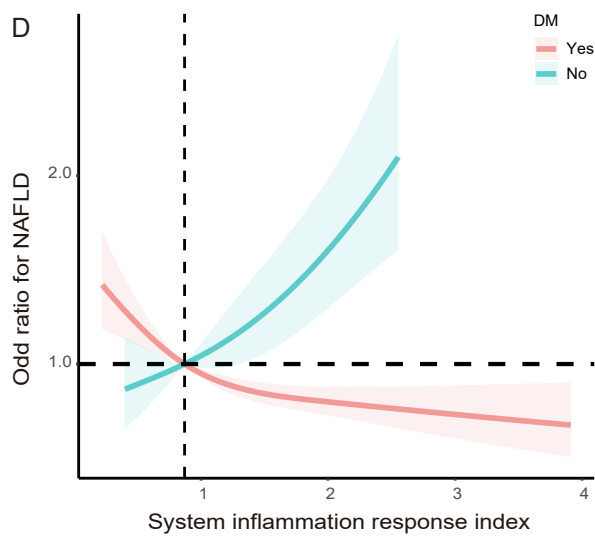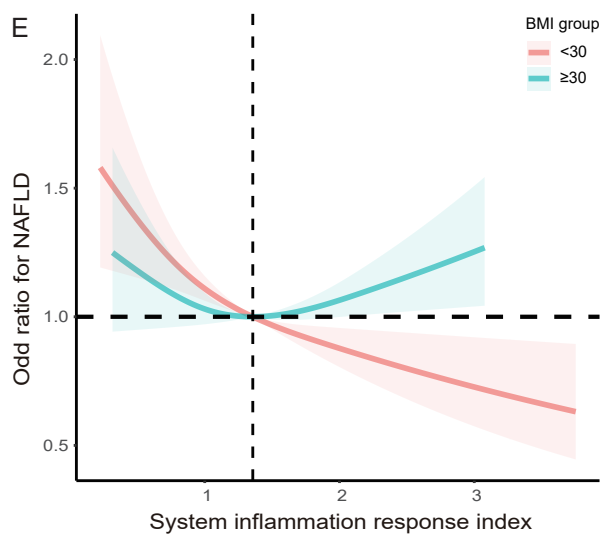

Supplement: Supplementary data 2 [file mmc2.pdf]
