# Supplementary material for: The association of systemic inflammatory biomarkers with non-alcoholic fatty liver disease: a large population-based cross-sectional study
Source: Prev Med Rep. 2023 Dec 7;37:102536. doi: 10.1016/j.pmedr.2023.102536 (PMC10767190; doi:10.1016/j.pmedr.2023.102536)
Supplement: Supplementary data 3 [file mmc3.docx]

| **Supplementary Table 1.** Stratification by age, sex, hypertension, DM, and BMI in the associations of SII index with prevalence of NAFLD in adults in the United States from NHANES 2017–2018 | | | | | | |
| --- | --- | --- | --- | --- | --- | --- |
| SII index | Q1 | Q2 | Q3 | Q4 | *P* for trend | *P* for interaction |
|  | OR (95%CI) | OR (95%CI) | OR (95%CI) | OR (95%CI) |  |  |
| Age |  |  |  |  |  | 0.122 |
| < 60 | 1.00 | 1.04 (0.58, 1.87) | 0.62 (0.34, 1.14) | 0.89 (0.50, 1.60) | 0.448 |  |
| ≥ 60 | 1.00 | 0.70 (0.30, 1.65) | 0.47 (0.19, 1.15) | 1.90 (0.76, 4.74) | 0.337 |  |
| Sex |  |  |  |  |  | 0.420 |
| Male | 1.00 | 0.91 (0.44, 1.87) | 0.56 (0.24, 1.29) | 1.07 (0.47, 2.44) | 0.834 |  |
| Female | 1.00 | 0.83 (0.44, 1.57) | 0.51 (0.28, 0.95) * | 1.00 (0.55, 1.82) | 0.835 |  |
| Hypertension |  |  |  |  |  | 0.596 |
| No | 1.00 | 0.96 (0.51, 1.83) | 0.62 (0.31, 1.22) | 1.12 (0.58, 2.17) | 0.941 |  |
| Yes | 1.00 | 0.84 (0.41, 1.71) | 0.49 (0.24, 0.99) * | 0.83 (0.42, 1.64) | 0.361 |  |
| DM |  |  |  |  |  | 0.235 |
| No | 1.00 | 0.87 (0.52, 1.46) | 0.46 (0.26, 0.79) * | 0.89 (0.52, 1.51) | 0.311 |  |
| Yes | 1.00 | 1.20 (0.33, 4.40) | 0.94 (0.28, 3.13) | 2.35 (0.74, 7.43) | 0.150 |  |
| BMI |  |  |  |  |  | 0.178 |
| < 30 kg/m^2^ | 1.00 | 0.96 (0.52, 1.76) | 0.37 (0.19, 0.72) ** | 0.92 (0.49, 1.73) | 0.260 |  |
| ≥ 30 kg/m^2^ | 1.00 | 0.97 (0.44, 2.15) | 0.83 (0.38, 1.80) | 1.24 (0.58, 2.65) | 0.568 |  |

Abbreviations: SII index, systemic immune inflammation index; NAFLD, non-alcoholic fatty liver disease; Q1, 50.000-291.652; Q2, 292.653-423.059; Q3, 423.060-603.132; Q4, 603.133-3250.715; **P* < 0.05; ***P* < 0.01; OR, odd ratio; CI, confidence interval. Analysis was adjusted for age, sex, race/ethnicity, education level, marital status, family poverty income ratio, the complication of hypertension, and diabetes mellitus, smoke status, and drink status, the complication of coronary heart disease, congestive heart failure, angina pectoris, heart attack, and stroke, body mass index, waist circumference, fast glucose, hemoglobin, high-sensitivity C-reactive protein, alanine aminotransferase, aspartate amino transferase, gamma-glutamyl transpeptidase, high-density lipoprotein-cholesterol, total cholesterol, triglyceride, blood urea nitrogen, uric acid, serum creatinine, and estimated glomerular filtration rate.
